# Spectral Clustering Community Detection Algorithm Based on Point-Wise Mutual Information Graph Kernel

**DOI:** 10.3390/e25121617

**Published:** 2023-12-03

**Authors:** Yinan Chen, Wenbin Ye, Dong Li

**Affiliations:** 1 Department of Computer Science and Technology, Shantou University, Shantou 515821, China; 2 School of Software Engineering, South China University of Technology, Guangzhou 510006, China; fhd573906@gmail.com (W.Y.); cslidong@scut.edu.cn (D.L.)

**Keywords:** community detection, spectral clustering, graph kernel, Bayesian inference, number of communities estimation

## Abstract

To address the problem that traditional spectral clustering algorithms cannot obtain the complete structural information of networks, this paper proposes a spectral clustering community detection algorithm, PMIK-SC, based on the point-wise mutual information (PMI) graph kernel. The kernel is constructed according to the point-wise mutual information between nodes, which is then used as a proximity matrix to reconstruct the network and obtain the symmetric normalized Laplacian matrix. Finally, the network is partitioned by the eigendecomposition and eigenvector clustering of the Laplacian matrix. In addition, to determine the number of clusters during spectral clustering, this paper proposes a fast algorithm, BI-CNE, for estimating the number of communities. For a specific network, the algorithm first reconstructs the original network and then runs Monte Carlo sampling to estimate the number of communities by Bayesian inference. Experimental results show that the detection speed and accuracy of the algorithm are superior to other existing algorithms for estimating the number of communities. On this basis, the spectral clustering community detection algorithm PMIK-SC also has high accuracy and stability compared with other community detection algorithms and spectral clustering algorithms.

## 1. Introduction

A complex network is an important cross-cutting research branch of computer science, statistical physics, and systems science and an indispensable tool for analyzing and studying interaction events in many real systems. Various kinds of networks and network-like systems are ubiquitous in real life, such as interpersonal networks [1], infectious disease networks, biological systems [2], and so on. In complex networks, the independent individuals in the system are usually referred to as nodes; the connections between these individuals are called edges, and clusters of closely connected nodes are called communities.

Community structure is an important feature of complex networks, and how to effectively perform community detection has attracted many scholars in various fields. Watts et al. first proposed a small-world network model by observing and studying the “small-world phenomenon” in real-world complex networks (known as the six-degree separation theory) [3]. Barabási et al. found that the degree distribution of real-world complex networks obeys a power-law distribution, indicating the scale-free nature of complex networks [4]. The study and analysis of the community structure help to uncover the laws of the dynamic evolution of the network, to find the weak points of the system, or to verify the corresponding functions of the system, which are of great importance to predict the future development of the network and its possible dynamic behavior.

Community detection is the process of revealing the latent community structure in complex networks, which has important applications in real life, such as mining social groups with common interests and similar social backgrounds in social networks for accurate content recommendation and building dynamics models in infectious disease networks to predict the development of epidemic trends and facilitate accurate measures for epidemic prevention and control.

Since Newman et al. proposed the classical GN algorithm [5], research on community detection algorithms has been enduring, and the spectral clustering algorithm based on spectral graph partitioning theory is one of the classical community detection algorithms. Through the comparative study of various community detection algorithms, including LPA-based, modularity-based, and information-entropy-based approaches, we found that, compared with these algorithms, spectral clustering-based algorithms can obtain higher accuracy on the condition that a suitable proximity matrix and the exact number of clusters are provided. However, traditional spectral-clustering-based algorithms can hardly handle community detection on complex networks, mainly for the following two reasons: First, most spectral clustering community detection algorithms cannot effectively work on complex networks with an unknown number of communities; second, limited by the singularity of structural features extracted from proximity matrixes, the generalization capability is not strong enough to effectively reflect the complex structural information in the network. Aiming at these two aspects, the BI-CNE algorithm and the PMIK-SC algorithm are proposed.

BI-CNE is a fast number of communities (or CN for short) estimation algorithm based on Bayesian inference. It is used to solve the problem that spectral clustering algorithms require prior knowledge about CN, while the traditional CN estimation methods are slow and inaccurate. The algorithm first performs a fast pruning reconstruction of the network, then performs Bayesian inference based on the degree-corrected stochastic block model, and finally obtains the CN estimated by Monte Carlo sampling. Meanwhile, in order to speed up the sampling process, the network reconstruction result is used as the initial state of sampling, and the overall sampling acceptance rate is improved by controlling the node transfer direction in the sampling procedure. Experiments show that the algorithm outperforms existing CN estimation algorithms.

Our preliminary research indicates that mutual information can effectively measure the relationships between communities in a network. Mutual-information-based community detection methods, such as MINC-NRL [6] and AMI-MLPA [7], can achieve accurate community detection. Similarly, the relationships between nodes can also be measured within an information-theoretic framework. We constructed a Laplacian kernel based on point-wise mutual information, referred to as the PMI kernel, and proposed a spectral clustering algorithm, PMIK-SC. The PMI kernel proves to be effective in addressing the problem that the proximity matrix used by the traditional spectral clustering algorithm cannot obtain the complete structure information for a specific network. This enhancement contributes to the accuracy of community detection tasks. Experiments show that the algorithm achieves better performance compared with state-of-the-art graph-kernel-based spectral clustering algorithms.

The rest of the article is organized as follows: Section 2 summarizes the current research progress in estimating the number of communities and graph-kernel-based spectral clustering algorithms. Section 3 introduces a large number of community estimation algorithms based on Bayesian inference. Section 4 derives the definition of a point-wise mutual information graph kernel and introduces the spectral clustering community detection algorithm based on the kernel. Section 5 conducts corresponding experiments to verify the effectiveness of the algorithm for the above two algorithms, and finally, Section 6 concludes the article with a summary of its contents.

## 2. Related Work

### 2.1. Traditional Algorithms for Estimating the Number of Communities

Most of the current spectral clustering community detection algorithms cannot efficiently partition complex networks with an unknown number of communities [8]. If information on the number of communities (CN) is included, the accuracy of these community detection algorithms can be greatly improved. Many scholars have proposed community structure estimation algorithms for this purpose, among which the most well-known is the modularity optimization method, which is essentially a class of algorithms that combines community size selection and performs community partitioning operations [9]. Modularity is an evaluation metric to measure the quality of community detection, proposed by Newman et al. in 2004 and updated by definition in 2006 [10]. The basic idea is that since there is no community structure in a random network, a community partition is better if it has a larger difference compared with a rule-based random network.

Although the modularity method is widely used, there are still two problems: first, for most of the real-world complex networks, the modularity value of ground-truth community partition does not reach optimal modularity; second, the modularity metric has a resolution limit problem [11], i.e., modularity-based methods cannot detect the very small communities in complex networks but prefer to divide the network into multiple large communities. Therefore, when estimating the CN in large-scale complex networks, such methods tend to obtain a smaller value, which may differ greatly from the actual CN.

The topological potential method is another common type of CN estimation algorithm. The basic idea is to extend the concept of potential and field in physics to complex networks and partition the network by high and low potential values to estimate the number. With the help of node topological potential, scholars propose a number of community estimation algorithms based on the hill-climbing method to search for local extrema. After calculating the topological potential of nodes in a complex network, the hill-climbing method traverses the nodes in the direction of rising potential and uses the local extremal points searched as community centers to obtain the number of communities.

The traditional hill-climbing method needs to calculate the topological potential of nodes, where the complexity of calculating the shortest path of any two nodes in the network is $O(n^{3})$, and the complexity of searching all the locally extremely potential nodes in the end is $O(n^{2})$, which is not reasonable, especially for large-scale networks. Secondly, the number of locally extremely potential nodes obtained by the algorithm is not necessarily the final CN because a community centroid may connect other community centroids and result in more than one community hiding at one local potential extreme point. To address this, scholars improved the algorithm by introducing a network concavity parameter by definition to search for potential local potential maximal nodes [12]. However, the improved algorithm can hardly handle the following two cases: First, multiple extreme points detected in the same community need to be identified and merged. Furthermore, the potential extreme points of different communities need to be split if they are covered by an edge to a larger extreme point. Therefore, the topological-potential-based algorithm for estimating the number of communities cannot be applied well to large-scale complex network analysis.

Neither modularity optimization algorithms nor heuristic algorithms, such as topological potential-based methods, can give satisfactory estimates of the number of communities. Therefore, some scholars tried to derive the actual number of communities by maximizing the approximation to the data likelihood from generative network graph models [13]. Among these methods, the most commonly used generative graph model is the stochastic block model (SBM) [14,15]. The estimation based on SBM mainly focuses on how to sample the probability space of the parameters and, therefore, needs to determine the likelihood of the parameters. Newman et al. proposed an algorithm for estimating the number of communities based on statistical inference using the SBM [8] to estimate the number of communities by Monte Carlo sampling. However, this method cannot be applied to large-scale networks due to computational speed limitations. Riolo et al. performed sampling acceleration optimization based on this method. They used an improved Chinese restaurant process to determine the parameters prior, which can be applied in large-scale networks, but the sampling speed is still far from satisfactory, and the estimation in large-scale networks is not completely accurate [16].

### 2.2. Graph-Kernel-Based Spectral Clustering Algorithm

To address the shortcomings of traditional clustering algorithms, especially the problem that the clustering algorithms are easily trapped in local optima, researchers proposed spectral clustering algorithms to solve the problem by introducing the spectral graph theory. In a spectral clustering algorithm, a suitable proximity matrix or similarity matrix needs to be constructed, which directly affects the final result of spectral clustering [17]. There are several methods to construct a proximity matrix; one of them is the kernel method. The core idea is to map the linearly inseparable data onto a linearly separable kernel space. The kernel function directly calculates the inner product of the mapping to represent the similarity among data without finding specific mapping relationships; the latter are usually difficult or impossible to solve.

The study of kernel methods in complex network structures falls into two main categories: graph embedding, which uses kernel functions to embed network structures into vector spaces, and graph kernels, which are mainly used as a way to measure the similarity of structures. Graph embedding yields a vectorized representation of the network structure, which is then processed by applying a vector-based kernel function. However, because the network data are downscaled to vector space, much structural information in the network cannot be preserved. In contrast, the graph kernel is directly oriented to the network structure data, and by defining a suitable kernel, the input network data are mapped from the original space to a high or infinite dimensional feature space, and the structured information in Hilbert space is preserved efficiently and completely. In the following, graph kernels or feature extraction methods based on the adjacency matrix, Laplacian matrix, and path length are briefly described, respectively.

The communicability kernel [18] is a graph kernel based on the adjacency matrix and belongs to the symmetric exponential diffusion kernel. In complex networks, the communicability between nodes is usually considered the shortest path length. The strategy of the communicability kernel is that a node can communicate with another node through all paths, but the longer the path, the lower the contribution to the node’s communication function.

The heat kernel [19,20] is a graph kernel based on the Laplacian matrix and belongs to a symmetric exponential diffusion kernel-like communicability kernel. Since the decay coefficient of the heat kernel is $\frac{(-t{)}^{k}}{k!}$, of which the decay rate is higher, some scholars have applied it to large-scale networks for community detection and obtained better results [20].

The commute time kernel [21] is a kind of path-length-based graph kernel. The commute time from the node $v_{i}$ to $v_{j}$ is usually defined as the expected time to start from the node $v_{i}$, randomly wander to node $v_{j}$ and back to node $v_{j}$. The commute time kernel is proved to be equal to the pseudo-inverse of the Laplacian matrix, and since the Laplacian matrix must have eigenvalues 0, L is irreducible, and the pseudo-inverse is usually computed using the Moore–Penrose generalized inverse.

All the graph kernels or methods mentioned above can extract some aspects of the structural features of graphs and combine them with community detection algorithms to analyze specific types of networks. However, limited by the singularity of the extracted structural features, the generalization ability is not strong, so it is necessary to design a graph kernel that can effectively reflect the complex structural information inside the graph and, at the same time, have strong generalization.

## 3. BI-CNE Algorithm

### 3.1. Network Pruning Reconstruction

For real-world complex networks, especially large-scale networks, it is hard to quickly estimate the CN due to the large number of nodes and edges. If the original network can be pruned by data pre-processing, such as removing unimportant nodes and edges, it will help to improve the effectiveness of subsequent analysis. In addition, if the original network can be quickly decomposed into several small connected graphs with sparse interconnections, the computational complexity can be greatly reduced, and the amount of information lost in this network splitting method is smaller compared with the node compression method (such as the Louvain algorithm [22]). Based on these two points, the original network will be pruned and reconstructed according to the idea of common neighbors before estimating the actual CN. First, consider the definition of a clique in a network: a clique is a subgraph of a network in which any node has a contiguous edge with all the remaining nodes. That is, a clique is a complete subgraph, but because the condition of a complete graph is too strict, the size of a clique in a real network is often not large enough to use a clique for a reasonable splitting of the original network. Therefore, we consider a relaxation of the condition of the corpus and use common neighbors to transform the condition of the complete graph as below: n−2 common neighbors exist between any two nodes in a complete graph of n nodes. The following section starts to consider pruning the nodes and edges in the network graph using the number of common neighbors.

**Figure 1:**
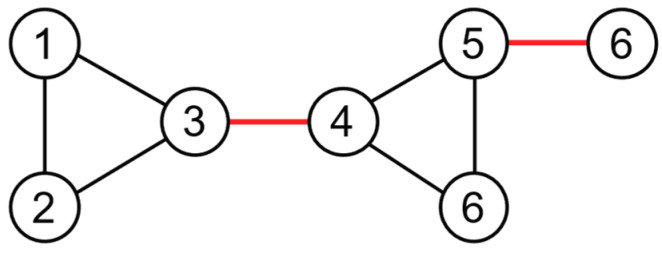
Before reconstruction.

**Figure 2:**
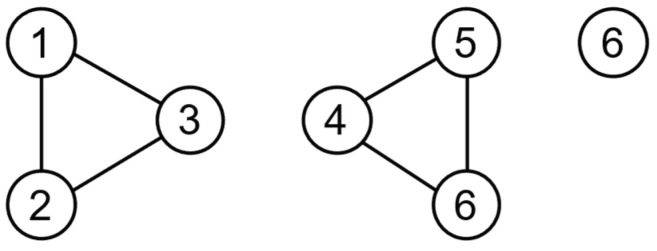
After reconstruction.

In a network graph, the number of common neighbors between two nodes is defined as the cutoff value of these two nodes. A cutoff value of 0 indicates that there are no common neighbors between nodes, which are usually reflected as peripheral nodes or inter-community bridge nodes of the network, as shown in Figure 1, where node 3 and node 4 are inter-community bridge nodes and node 5 and node 6 are peripheral nodes. At this point, if the edges with cutoff values less than 1 are removed from the network, the original network will be decomposed into three connected graphs, including two n=3 complete subgraphs and one stray node, as shown in Figure 2.

**Figure 3:**
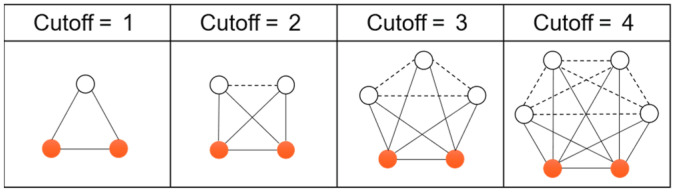
Connection status of solid nodes under different cutoffs.

To use the cutoff value to relax the condition of the clique, it is necessary to determine the appropriate range of cutoff values. The node connectivity in the network at cutoff in [1,4] is given below, as shown in Figure 3. The solid nodes are the observed objects, and the hollow nodes are the common neighbors of the two solid nodes. The solid lines indicate the real connected edges, and the dashed lines indicate the connected edges that need to be added to form a clique. It can be found that as the cutoff value increases, the proportion of dashed edges to be replenished is larger, the relaxation condition is stricter, and the degree of network decomposition is higher. Assuming that the cutoff value is k, the number of real edges in the network is 2k+1, and the number of supplementary edges is k(k−1)/2, and when k=6, the number of supplementary edges is higher than the number of real edges. In other words, the number of supplementary edges required to restore the edges from two real nodes to a clique in a local network exceeds the number of existing edges. Moreover, real-world complex networks are usually sparse, i.e., the average degree is low, and partitioning the network with a high cutoff value will result in a large number of free nodes, which is not consistent with the original idea of network reconstruction. Therefore, the network should be pruned by a reasonable value of k selected in the range of [0,6].

### 3.2. Bayesian Inference

**Figure 4:**
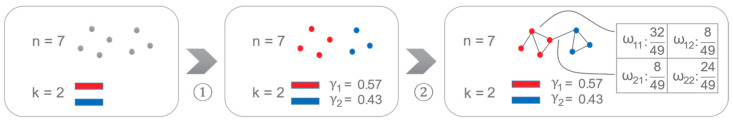
Stochastic block model.

After completing the pruning reconstruction of the network, the BI-CNE algorithm uses the degree-corrected stochastic block model to fit real-world complex networks, and the generation process of a random network by the original stochastic block model is shown in Figure 4. Firstly, given the number of nodes n and the number of communities k of the network, nodes are randomly assigned to k communities with community assignment probability $\gamma =\{\gamma_{r}|r\in [1,\ldots ,k],\;\sum_{r=1}^{k}\gamma_{r}=1\}$. In turn, the second step randomly assigns nodes to k communities according to the connected edge probability matrix ω connects nodes, i.e., the probability that there exists a connected edge between node $v_{i}$ located in community r and node $v_{j}$ located in community s is $\omega_{rs}$. In an undirected graph, $\omega_{rs}=\omega_{sr}$. Since the probability of connecting edges between nodes in the process of connecting edges depends only on the community to which they belong, and the specific node attributes have no effect on the probability of connecting edges, the original stochastic block model can only fit networks whose degrees obey Poisson distributions. This is the reason why the original stochastic block model for CN estimation or community detection does not give good results in real-world networks.

**Figure 5:**
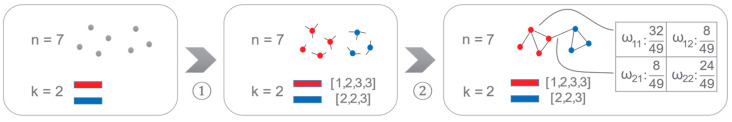
Degree-corrected stochastic block model.

The degree-corrected stochastic block model makes it possible to fit a network with an arbitrary degree distribution by differentiating the node connectivity probabilities, and the process of generating a random graph is shown in Figure 5. The main difference between it and the original stochastic block model is that in the second step, the connectivity probability between nodes depends not only on the community to which they belong but also on the degree distribution of the nodes, i.e., the connectivity probability between a node $v_{i}$ located in community r and a node $v_{j}$ located in community s is $\theta_{i}\theta_{j}\omega_{rs}$. As the degree sequence of the four nodes of the red community in Figure 5 is [1,2,3,3], which represents the expected degree of each node, the ratio of the edges connected into the red community to the nodes in the community will be controlled as 1:2:3:3. Since the size of each community node is not consistent, the parameter θ within each community needs to be normalized.

On the basis of the degree-corrected stochastic block model, consider the inverse process of its generation of random networks, i.e., deriving the number of real communities of the network backward from the existing network structure. The specific derivation process is given below. Given an undirected network G=(V,E), the number of nodes is |V|=n, and the number of edges is |E|=m. Let the adjacency matrix be A, then $A_{ij}$ denotes the number of connected edges between nodes i and j, and the diagonal element $A_{ii}$ is equal to twice the number of self-looping edges of node i. A community partition is defined as $g=\{g_{i}|i\in [1,\ldots ,n]\}$, where $g_{i}$ denotes the number of the community to which node i belongs. The connected edges between nodes $v_{i}$ and $v_{j}$ located in community r and community s, respectively, obey a Poisson distribution with mean $\theta_{i}\theta_{j}\omega_{g_{i},g_{j}}$, and $\left\{\theta_{i}\right\}$ and $\left\{\omega_{rs}\right\}$ are the model parameters. For computational convenience, the parameters θ are constrained and normalized:

(1) $$\frac{1}{n_{r}}\sum_{i=1}^{n}\theta_{i}\delta \left(r,g_{i}\right)=1$$

where $n_{r}$ is the number of nodes within the denoted community r, $\delta (r,g_{i})$ is the Kronecker function defined as:

(2) $$\delta \left(s,t\right)=\left\{\begin{array}{c} 1,\;if\;s=t\\ 0,\;otherwise \end{array}\right.$$

Given the number of communities k, community partition g, parameters θ, ω, the probability of generating the specified network with adjacency matrix A is:

(3) $$\begin{array}{cc} P\left(A|\omega ,\theta ,g,k\right) & =\prod_{i<j}{\left(\theta_{i}\theta_{j}\omega_{g_{i}g_{j}}\right)}^{A_{ij}}e^{-\theta_{i}\theta_{j}\omega_{g_{i}g_{j}}}\\ & \times \prod_{i}{\left(\frac{1}{2}\theta_{i}^{2}\omega_{g_{i}g_{i}}\right)}^{A_{ii}/2}e^{-\theta_{i}^{2}\omega_{g_{i}g_{i}}/2} \end{array}$$

where the first concatenated part represents the inter-community edge probability between nodes, and the second concatenated part represents the edge probability between nodes within the same community. Substituting Equation (1) into Equation (3) and neglecting the constant multiplier, we obtain:

(4) $$P\left(A|\omega ,\theta ,g,k\right)=\prod_{i}\theta_{i}^{d_{i}}\prod_{r<s}\omega_{rs}^{m_{rs}}e^{-n_{r}n_{s}\omega_{rs}}\prod_{r}\omega_{rr}^{m_{rr}}e^{-n_{r}^{2}\omega_{rr}/2}$$

where $d_{i}$ denotes the degree of node $v_{i}$ and $m_{rs}$ denotes the total number of connected edges between community r and community s. Since the intermediate parameters θ and ω are irrelevant to the problem modeling, their priori selection and integral elimination are considered. Here, the reference [16] for the priori selection of parameters θ and ω yields the final likelihood function as shown in Equation (5), where $\kappa_{r}=\sum_{i}d_{i}\delta_{r,g_{i}}$ is the sum of the degrees of all nodes within the community r.

(5) $$P\left(A|g,k\right)=\prod_{r}\;n_{r}^{\kappa_{r}}\frac{\left(n_{r}-1\right)!}{\left(n_{r}+\kappa_{r}-1\right)!}\times \prod_{r<s}\;\frac{m_{rs}!}{{\left(pn_{r}n_{s}+1\right)}^{m_{rs}+1}}\prod_{r}\;\frac{m_{rr}!}{{\left(\frac{1}{2}pn_{r}^{2}+1\right)}^{m_{rr}+1}}$$

On the basis of determining the parameter prior and obtaining the likelihood function, the Bayesian model can be used for the task of inferring the number of communities k. Given the network adjacency matrix A, the probability distribution P(g,k∣A) is shown in Equation (6).

(6) $$P\left(g,k|A\right)=\frac{P\left(g,k\right)P\left(A|g,k\right)}{P\left(A\right)}$$

where the likelihood function P(A|g,k) has been determined. The joint probability distribution P(g,k) can be calculated using Equation (7) as in [16]. At this point, the Bayesian inference process for the degree-corrected stochastic block model has been completed, providing a theoretical basis for Monte Carlo sampling in the next subsection.

(7) $$P\left(g,k\right)=(n-2{)}^{-k}\prod_{r=1}^{k}n_{r}!\;$$

### 3.3. Monte Carlo Sampling

After completing the priori derivation of the key parameters, the complete expression of the conditional probability P(g,k|A) is determined by ignoring the observed data P(A). The posterior probability P(k|A) of the number of communities k can be obtained by counting all community partition cases g, so that the most probable number of communities k can be deduced for the purpose of estimation. However, since the total number of all possible cases of partitions is $k^{n}$, which are impossible to exhaustively traversed. Here, the Monte Carlo method is introduced to sample these cases. The pair (k,g) is considered as the “state” of the network to be sampled, and k is counted during sampling; finally, the k corresponding to the maximum value of P(k|A) is the estimated number of communities. The sampling process consists of two main types of sampling steps:

- Move node $v_{i}$ from community r to an existing community s, then
  (8) $$k=\left\{\begin{array}{c} k-1,\;if\;r=\left\{v_{i}\right\}\\ k,\;otherwise \end{array}\right.$$
- Move node $v_{i}$ to a new community, then k=k+1.

An effective Monte Carlo sampling algorithm needs to satisfy ergodicity and detailed balance. Ergodicity requires that each state of the system be accessible to each other through a finite sequence of Monte Carlo steps. For this reason, the above process of moving a single node from one community to another satisfies this condition. And for careful equilibrium needs to be satisfied: the ratio $R(g,k\to g^{\prime },k^{\prime })$ of traversing from the current state (g,k) to another state $\left(g^{\prime },k^{\prime }\right)$ and the ratio $R(g^{\prime },k^{\prime }\to g,k)$ of returning back must satisfy:

(9) $$\frac{R\left(g,k\to g^{\prime },k^{\prime }\right)}{R\left(g^{\prime },k^{\prime }\to g,k\right)}=\frac{P\left(g^{\prime },k^{\prime }\right)}{P\left(g,k\right)}\times \frac{P\left(A|g^{\prime },k^{\prime }\right)}{P\left(A|g,k\right)}$$

A traditional acceptance/rejection pattern is used in each step, where the move operation is performed with probability π, and the operation is accepted with probability α.

(10) $$\frac{R\left(g,k\to g^{\prime },k^{\prime }\right)}{R\left(g^{\prime },k^{\prime }\to g,k\right)}=\frac{\pi \left(g,k\to g^{\prime },k^{\prime }\right)}{\pi \left(g^{\prime },k^{\prime }\to g,k\right)}\times \frac{\alpha \left(g,k\to g^{\prime },k^{\prime }\right)}{\alpha \left(g^{\prime },k^{\prime }\to g,k\right)}$$

The final Monte Carlo sampling process is as follows:

(1) Initialization: Disorder the nodes and assign them to the given maximum $k_{max}$ communities; note that there is no empty community here.
(2) Sampling: Execute Operation 1 with probability 1−1/(n−1) or Operation 2 with probability 1/(n−1).
  - Operation 1: Randomly select communities r,s. Randomly select a node $v_{i}$ from community r and move it to community s. If node $v_{i}$ is the last node of community r, then delete community r and renumber the communities, and that makes k=k−1.
  - Operation 2: Randomly select a community r. Randomly select a node $v_{i}$ from community r and move it to a new empty community k+1. If node $v_{i}$ is the last node of community r, this operation is rejected, and k remains unchanged. Otherwise, it makes k=k+1.
(3) Accept the operation: The operation in Step 2 will be accepted following the acceptance probability:
  (11) $$\alpha \left(g,k\to g^{\prime },k^{\prime }\right)=min\left(1,\frac{P\left(A|g^{\prime },k^{\prime }\right)}{P\left(A|g,k\right)}\right)$$
(4) Repeat steps 2 and 3.

### 3.4. Sampling Acceleration

Experiments on the Monte Carlo sampling proposed in the previous section reveal that there are some points that can be optimized in the initialization step and the sampling Operation 1 of the sampling process. For the initialization in Step (1), nodes are randomly assigned to $k_{max}$ communities, and there is a high probability that nodes assigned to the same community are not connected to each other, so the subsequent sampling process requires a large number of iterations to reach a more reasonable community partition state. To address this problem, the network reconstruction method in Section 3.1 can be applied to assign nodes belonging to the same connected clique after reconstruction to the same initial community and all free nodes to their own separate communities.

For Operation 1, i.e., randomly selecting communities r, s and randomly moving a node $v_{i}$ from community r to community s, the problem is the low sampling acceptance rate. Since the selection of community s is random and only a few communities have edges linked to node $v_{i}$, this node-transfer community operation will be rejected with high probability. This leads to a slow rate of sampling, so it is necessary to control the selection of community s. First, if a node’s community transfer operation is accepted, it means that the new partition state of the node’s community is more likely to reflect the original network topology information than that before the change. Therefore, in the case that the node $v_{i}$ is not connected to the other nodes of the original community r, it is considered that there is a high probability that it will transfer to any other community, so we still select the community s equiprobably; while in the case that node $v_{i}$ is connected to the other parts of community r, a certain weight is assigned to each community according to the nodes’ connectedness to determine the probability of selecting community s. The weight of the node $v_{i}$ to transfer from the community r to community s is calculated as shown in Equation (12):

(12) $$w\left(g_{i}^{\prime }=s|g_{i}=r\right)=\sum_{t}\beta_{it}\frac{m_{ts}+\alpha }{n_{t}+\alpha \cdot k}$$

where $m_{rs}$ denotes the number of connected edges between community r and community s, $n_{t}$ denotes the total number of nodes in community t, and k is the number of current communities. The communities that are connected to the node $v_{i}$ are called node-neighboring communities, and the control parameter α ensures that each community has a certain probability of being selected, regardless of whether the number of connected edges $m_{ts}$ of the community with node-neighboring communities is 0, and α can be set to 1. $\beta_{it}$ denotes the weight of the number of contiguous edges between node $v_{i}$ and community t in its own degree, which is calculated as:

(13) $$\beta_{it}=\frac{\sum_{j}A_{ij}\delta \left(g_{i},t\right)}{d_{i}}$$

where $A_{ij}$ is the number of edges connecting node $v_{i}$ and node $v_{j}$. $\delta (g_{i},t)$ is the Kronecker function. $d_{i}$ is the degree of node $v_{i}$. The main idea of the community transfer weight formula Equation (12) is that the weight of node $v_{i}$ transferring to community s will be greater if:

- Node $v_{i}$ is more closely connected to its neighboring communities;
- Community s is more closely connected to the neighboring communities of node $v_{i}$;
- The size of the community s is smaller.

The final node transfer probability is calculated as shown in Equation (14).

(14) $$P\left(g_{i}^{\prime }=s|g_{i}=r\right)=\frac{w\left(g_{i}^{\prime }=s|g_{i}=r\right)}{\sum_{t}w\left(g_{i}^{\prime }=t|g_{i}=r\right)}$$

**Figure 6:**
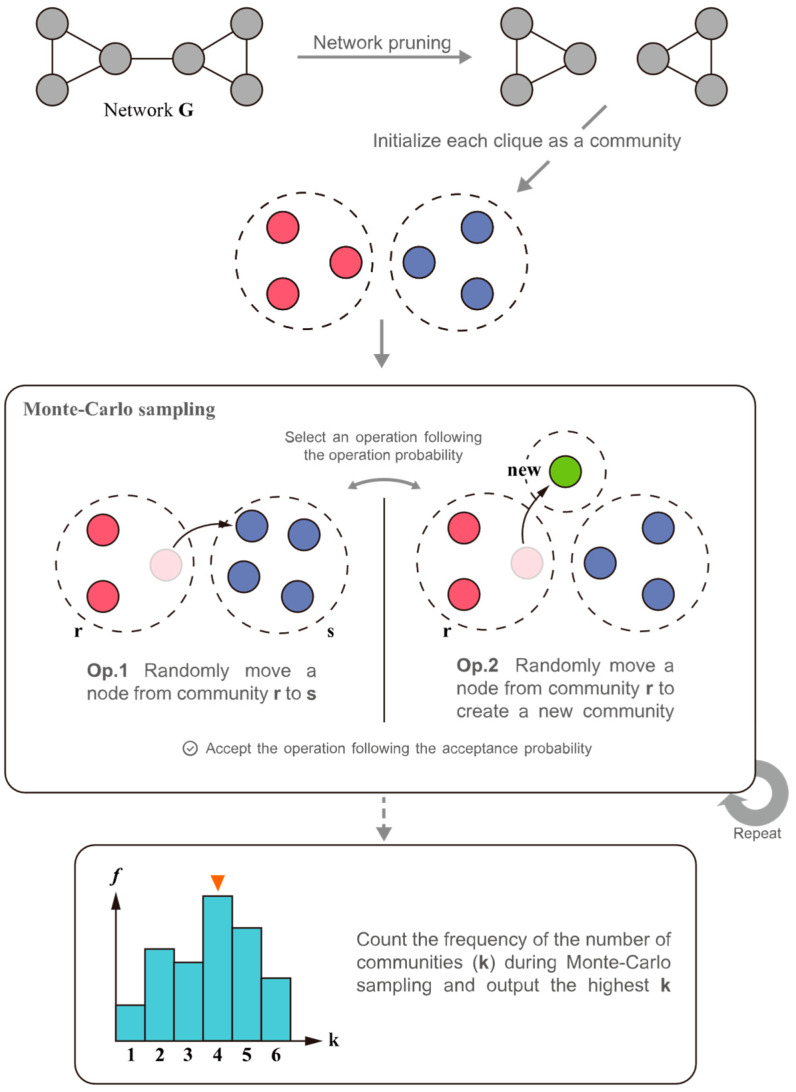
Main flow of BI-CNE.

So far, the derivation and algorithm design of a large number of community estimation algorithms based on Bayesian inference have been completed. Figure 6 shows the main procedure of the algorithm. The specific experimental results are shown in Section 5.2.

## 4. PMIK-SC Algorithm

### 4.1. PMI-Kernel Derivation

One of the key problems in spectral clustering algorithms is how to construct a suitable proximity matrix, and graph kernel is one of the methods to construct such a matrix. For structural information, graph kernels of different construction methods can extract different structural features of networks, such as kernels based on the shortest path, which may give more weight to short edges in the network, while kernels based on sub-tree structure can obtain richer information about the graph structure but also have the defect of path backtracking, etc. Currently, many graph kernels are defined based on R-convolution theory for construction, but such kernels have three drawbacks:

- A large amount of structural information in non-isomorphic subgraphs is ignored.
- The positions of isomorphic sub-structures in the original network cannot be reflected by the kernels.
- The kernels only deal with small-size sub-structures, which cannot fully reflect the structural information of the network.

From the perspective of information theory, we introduce point-wise mutual information (PMI) and design the PMI-Kernel based on the exponentially decaying diffusion model. Point-wise mutual information is used to measure the information-theoretical correlation between two variables.

Compared to R-convolution-based kernels, PMI-based kernels have the following features: First, instead of directly extracting the features of nodes, it aims to reveal the correlation between nodes. This transforms the community detection problem into a node clustering problem and minimizes the information loss during the clustering process as much as possible. In addition to this, the point-wise mutual information matrix constructed based on the infinite-order transition probability matrix can not only express the local information of each node’s neighborhood but also retain the global information of the target network.

Therefore, graph kernels based on PMI avoid the drawbacks of R-convolution-based kernels. PMI-based kernels no longer need to consider the isomorphism and position of the substructure, and the neighborhood substructure information of a specific node is directly stored in the corresponding row of the PMI matrix in the form of multiple-order accumulation. In addition, since the PMI matrix is constructed based on the infinite-order transition probability matrix, the global information of the network is preserved, so PMI-based kernels can more fully reflect the structural information of a network.

For a pair of discrete random variables x and y, the point-wise mutual information is defined as the logarithm of the ratio of the product of the joint probability distribution and the marginal probability distribution, as shown in Equation (15):

(15) $$pmi\left(x;y\right)=\log\;\frac{p\left(x,y\right)}{p\left(x\right)p\left(y\right)}$$

The value range of PMI is:

(16) $$-\infty \leq pmi\left(x;y\right)\leq min\left[-log\;p\left(x\right),-log\;p\left(y\right)\right]$$

The PMI value indicates the correlation between two random variables. If x and y are independent, pmi(x;y) is 0; if there is a negative correlation between x and y, pmi(x;y) will be negative.

In order to apply PMI to the network structure, it is necessary to select suitable probabilities as edge probabilities and joint probabilities in Equation (15). First, consider the first-order transfer probability matrix, $P_{1}$:

(17) $$P_{1}=D^{-1}A$$

where D and A are, respectively, the degree matrix and the adjacency matrix of the network. Experiments show that if the first-order transfer probability matrix is used to calculate the PMI values of a network with a lower average degree, the accuracy of community detection is low. This is due to the fact that global information cannot be obtained by the first-order transfer probability matrix in a sparse network. To address this problem, the exponentially decaying diffusion model based on the transfer probability matrix is introduced to calculate the infinite-order transfer probability matrix P of a specific network, as shown in Equation (18).

(18) $$P=\sum_{k=0}^{\infty }e^{-k}{P_{1}}^{k}=\sum_{k=0}^{\infty }{\left(\frac{P_{1}}{e}\right)}^{k}={\left[I-\frac{P_{1}}{e}\right]}^{-1}$$

The exponentially decaying diffusion model follows the principle that the influence between nodes decays with the increase in their distance in the network. Such information on pairwise influence contributes to finding the closely connected nodes in a network, and that is exactly the goal of community detection.

The sum of the elements in each row of matrix P is not necessary 1; here, it needs to be row normalized by:

(19) $$\tilde{P}=D_{P}^{-\frac{1}{2}}PD_{P}^{-\frac{1}{2}}$$

where $D_{P}$ is a diagonal matrix in which elements on the diagonal are the sum of the elements of the corresponding row in matrix P, and other elements 0, as shown in Equation (20):

(20) $$D_{P}\left(i,i\right)=\sum_{j}P\left(i,j\right)$$

In the normalized transfer probability matrix $\tilde{P}$, each element $\tilde{P}(i,j)$ is equal to the sum of the 1st, 2nd, …, and h-th order transfer probabilities from node i to node j. Then, the elements of the PMI matrix $M_{PMI}$ can be calculated by substituting each $\tilde{P}(i,j)$ into Equation (15):

(21) $$M_{PMI}\left(i,j\right)=\log\frac{\frac{\tilde{P}\left(i,j\right)}{V_{\tilde{P}}}}{\frac{\tilde{P}\left(i,\cdot \right)}{V_{\tilde{P}}}\frac{\tilde{P}\left(\cdot ,j\right)}{V_{\tilde{P}}}}=\log\frac{\tilde{P}\left(i,j\right)\cdot V_{\tilde{P}}}{\tilde{P}\left(i,\cdot \right)\tilde{P}\left(\cdot ,j\right)}$$

where $V_{\tilde{P}}$ denotes the flux of the $\tilde{P}$ matrix, i.e., the sum of the values of all elements of matrix $V_{\tilde{P}}$. $\tilde{P}(i,\cdot )$ and $\tilde{P}(\cdot ,j)$, respectively, denote the sum of all elements of a row and that of a column in matrix $\tilde{P}$.

Note that the PMI matrix obtained at this point cannot be directly used as a proximity matrix or kernel matrix for spectral clustering. That is because:

- The matrix is not symmetric since the transfer probability from node i to node j is not necessarily equal but determined by the degree of the two nodes and all possible paths starting from node i and node j.
- From the range of PMI values: [−∞, min[−log p(x),−log p(y)]], we know that there may be negative elements in the matrix.

To address the above two issues, the following equation is first used to symmetrize the matrix:

(22) $$M_{PMI}^{\prime }=\frac{1}{2}\left(M_{PMI}+M_{PMI}^{T}\right)$$

Then, for the negative values, the elements of matrix $M_{PMI}^{\prime }$ are normalized to 0∼1 in order to retain the original structural information of networks with different average degrees, as shown in Equation (23):

(23) $$K_{PMI}\left(i,j\right)=\frac{M_{PMI}^{\prime }\left(i,j\right)-min\left(M_{PMI}^{\prime }\right)}{max\left(M_{PMI}^{\prime }\right)}$$

### 4.2. Implementation

Based on the BI-CNE algorithm and PMI-Kernel, the implementation of the spectral clustering community detection algorithm based on the point-wise mutual information graph kernel (PMIK-SC) will be described in detail in terms of the number of communities, graph reconstruction, and graph cut criterion.

#### 4.2.1. Number of Communities

For the input requirement of the number of communities k for the PMIK-SC algorithm, the BI-CNE algorithm proposed in Section 3 is used to quickly estimate the number of communities k for the network. This is conducted by Monte Carlo sampling various community partition states of the network and selecting the k value corresponding to the maximum value of P(k∣A) as the number of communities; in short, the k value with the most frequency. Since the time complexity of the BI-CNE algorithm is only O(kn), and the number of iterations required for the algorithm to converge is greatly reduced by the acceleration of sampling, the number of communities k can be quickly obtained as the input to the PMIK-SC algorithm.

#### 4.2.2. Graph Reconstruction

**Figure 7:**
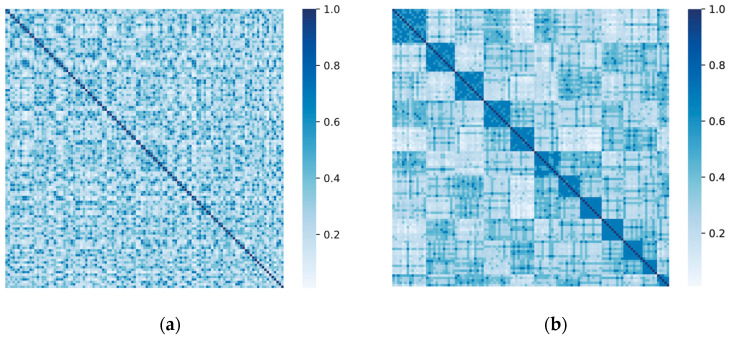
PMI matrix of Football network before and after re-arrangement by clustering. (**a**) Original PMI matrix. (**b**) Clustered PMI matrix.

The PMI-Kernel matrix $K_{PMI}$ derived in the previous section is used as the proximity matrix for spectral clustering. For small-scale and medium-scale networks, the infinite-order transfer probability matrix can extract rich topology information from the network, and the similarity between any two nodes can be represented by the corresponding element values in the PMI-Kernel matrix. This helps the spectral clustering algorithm better delineate the community structure, as shown in Figure 7. In contrast, for large-scale sparse networks, the computational cost can be reduced by limiting the influence range of nodes. For example, if the influence range of each node is set to l hops, the topological structural information of the network can be extracted by an l-order transfer probability matrix. This can be simply implemented by replacing ∞ by l in Equation (18). The impact of replacing the infinite-order transition probability matrix with an l-order transfer probability matrix is experimentally analyzed, as in Section 5.3.1.

To reconstruct the graph for spectral clustering using the PMI-Kernel matrix $K_{PMI}$, a distance matrix S is constructed first by using the proximities to distances formula [23]:

(24) $$S\left(i,j\right)=\frac{1}{2}\left(K_{PMI}\left(i,i\right)+K_{PMI}\left(j,j\right)\right)-K_{PMI}\left(i,j\right)$$

where S(i,j) is the elements of S. The distance matrix is then used to construct a weight adjacency matrix W, using the K-nearest neighborhood (*KNN*) construction:

(25) $$W\left(i,j\right)=W\left(j,i\right)=\left\{\begin{array}{c} \exp\;\left(-\frac{S\left(i,j\right)}{2\sigma^{2}}\right),\;if\;i\in KNN\left(j\right)\;or\;j\in KNN\left(i\right)\\ 0,\;\;otherwise \end{array}\right.$$

where *KNN*(i) denotes the $K_{N}$ neighbor nodes of node i, in which the distances between nodes are measured using S. σ is the variance of the Gaussian distribution, which is set to 1.0 on implementation.

Finally, a graph cut criterion is used to partition the reconstructed graph to obtain the result of spectral clustering.

#### 4.2.3. Graph Cut Criterion

The normalized cut (NCut) is selected as the cut criterion to partition the reconstructed graph. This criterion can measure both the degree of similarity between nodes within the same community and the degree of difference between nodes in different communities, which performs better on spectral clustering compared with other graph-cut criteria. Note that the NCut criterion requires an eigendecomposition and eigenvector clustering of a symmetric normalized Laplacian matrix $L_{sym}$, which is constructed by the following definition:

(26) $$WL_{sym}={D_{W}}^{-\frac{1}{2}}L{D_{W}}^{-\frac{1}{2}}$$

where $D_{w}$ is the degree matrix of W in which elements on the diagonal are the sum of the elements of the corresponding row in matrix W, and other elements 0, as: $D_{W}(i,i)=\sum_{j}W(i,j)$. L is the Laplacian matrix constructed by $L=D_{W}-W$.

On this basis, the community partition results are obtained by calculating the eigenvectors corresponding to the smallest k eigenvalues of $L_{sym}$, and finally, clustering using the k-means algorithm.

The procedure of the algorithm is as follows:

Input: adjacency matrix A of network G, the number of communities k

(1) Calculate the first-order transfer probability matrix $P_{1}=D^{-1}A$ and the infinite-order transfer probability matrix P according to Equation (18).
(2) Calculate the PMI matrix $M_{PMI}$, according to Equation (21).
(3) Symmetrize and normalize $M_{PMI}$ to obtain the PMI-Kernel matrix $K_{PMI}$.
(4) Calculate the distance matrix S using Equation (24).
(5) Reconstruct the network based on S by Equation (25) and obtain the weight adjacency matrix W.
(6) Construct the symmetric normalized Laplacian matrix $L_{sym}$ using Equation (26).
(7) Eigendecompose the Laplacian matrix $L_{sym}$ to obtain the first k smallest eigenvalues and the corresponding eigenvectors to form the feature matrix.
(8) Perform k-means clustering on the row vectors of the feature matrix to obtain the final community partitioning result g.

The pseudo-code of the algorithm is shown in Algorithm 1.


**Algorithm 1.** PMIK-SC algorithm**Require:** Adjacency matrix A, number of communities k
**Ensure:** Community partition g1:     $D\leftarrow diag(\sum_{j}A_{ij})$
2:     $P_{1}\leftarrow D^{-1}A$
3:     $P\leftarrow {\left[I-P_{1}/e\right]}^{-1}$
4:     $D_{P}\leftarrow diag(\sum_{j}P_{ij})$
5:     $\tilde{P}\leftarrow {D_{P}}^{-\frac{1}{2}}P{D_{P}}^{-\frac{1}{2}}$
6:     **for**  i,j←0
**to** n **do**7:       $M_{PMI}\left(i,j\right)\leftarrow \log\;\left(\left(\tilde{P}\left(i,j\right)V_{\tilde{P}}\right)/\left({\tilde{P}}_{i\cdot }\left[i\right]{\tilde{P}}_{\cdot j}\left[j\right]\right)\right)$
8:     **end for**9:     $K_{PMI}\leftarrow$ symmetrize and normalize $M_{PMI}$
10:   S← proximities_to_distances($M_{PMI}$)11:   **for**
i,j←0 **to**
n **do**12:     **if**
i **in** KNN(j) **or** j
**in** KNN(j) **then**13:       $W(i,j)\leftarrow exp\;(-S(i,j)/2\sigma^{2})$
14:     **else**15:       W(i,j)←0
16:     **end if**17:   **end for**18:   $D_{W}\leftarrow diag(\sum_{j}W_{ij})$
19:   $L\leftarrow D_{W}-W$
20:   $L_{sym}\leftarrow {D_{w}}^{-\frac{1}{2}}L{D_{w}}^{-\frac{1}{2}}$
21:   $values,vectors\leftarrow eig(L_{sym})$
22:   sort_ascending(values,vectors)
23:   g←k−means(vectors[:k],n_clusters=k)
24:   **return** community partition result g



## 5. Experiment

### 5.1. Preparation of the Experiments

#### 5.1.1. Datasets

The experiments are conducted on both real-world network datasets and the LFR synthetic network datasets [24]. The main properties of them are shown in Table 1 and Table 2, respectively, where n is the number of nodes, m is the number of edges, K is the ground-truth number of communities, d is the average degree of nodes, and μ is the mixing parameter for LFR networks.

Among them, L1–L4 have the same scale, which is used to compare the accuracy of BI-CNE and PMIK-SC algorithms under different mixing parameters (μ). L5–L9 are generated using the same d and μ, but with different numbers of nodes, to measure the performance of PMIK-SC under different network scales.

#### 5.1.2. Evaluation Indexes

For the CN estimation, the ground-truth number of communities is used as the evaluation index. It is better that the CN estimated by the algorithms is closer to the ground truth. For the community detection task, the evaluation indexes are the normalized mutual information (NMI) and modularity (Q). NMI [25] can be used to measure the difference between the partitions obtained by the community detection algorithm and the ground-truth partitions. The larger the *NMI* value, the closer the community partition result is to the ground-truth partitions.

(27) $$NMI=\frac{-2\sum_{i=1}^{C_{A}}\;\sum_{j=1}^{C_{B}}\;C_{ij}\log_{2}\;\left(C_{ij}N/C_{i.}C_{.j}\right)}{\sum_{i=1}^{C_{A}}\;C_{i.}\log_{2}\;\left(C_{i.}/N\right)+\sum_{j=1}^{C_{B}}\;C_{.j}\log_{2}\;\left(C_{.j}/N\right)}$$

where A represents the ground-truth partition, and B represents the partition obtained by a community detection algorithm; $C_{A}$ and $C_{B}$ represent the numbers of communities of A and B respectively; C is a confusion matrix, in which the element $C_{ij}$ represents the number of nodes both in community i in A and in community j in B. $C_{i.}$ and $C_{.j}$ are respectively the sum of the elements in the ith row and in jth column of C, and N represents the total number of nodes of the network.

Modularity is an evaluation index to measure the quality of partition given a partitioned network [5,10], which can be calculated by Equation (28):

(28) $$Q=\frac{1}{2m}\sum_{uv}\left[A_{uv}-\frac{k_{u}k_{v}}{2m}\right]\delta \left(c_{u},c_{v}\right)$$

where u,v denotes a pair of nodes, and $k_{u}$, $k_{v}$ are the degrees of them. m denotes the total number of edges in the network. A is the adjacency matrix of the network, in which the element $A_{uv}$ denotes the number of edges connecting node u and node v. $c_{u}$, $c_{v}$ denote the communities to which nodes u and v belong. $\delta (c_{u},c_{v})$ is the Kronecker delta defined as Equation (2).

Conductance has been widely used in the study of graph cuts before it was applied to community detection [26,27]. For a cluster c, conductance is defined as:

(29) $$f(c)=\frac{m_{c}^{ext}}{2m_{c}^{ext}+m_{c}^{int}}$$

where $m_{c}^{int}$ is the number of intra-cluster edges of cluster c, and $m_{c}^{ext}$ is the external edges link the cluster to other parts of the network. Here, we use the average conductance (AC) of all communities to evaluate the result of community detection:

(30) $$\Phi_{c}=\underset{c\in C}{\mathrm{avg}}\;f(c)$$

Intra-cluster density [28] is used to measure the density of edges within a cluster, which is defined as:

(31) $$\delta_{int}(c)=\frac{m_{c}}{n_{c}(n_{c}-1)/2}$$

where $n_{c}$ and $m_{c}$ is respectively the number of nodes and edges of cluster C. The denominator denotes the number of possible edges within the cluster, which is equal to $n_{c}(n_{c}-1)/2$.

Similarly, we use the average intra-cluster density (AICD) to measure the quality of a partition, which is exactly the average of the intra-cluster density of all communities in a partition:

(32) $$\Delta_{int}(C)=\underset{c\in C}{\mathrm{avg}}\;\delta_{int}(c)$$

#### 5.1.3. Hardware Information

The experiments are conducted single-threaded on a PC with the following technical details in Table 3.

### 5.2. BI-CNE Algorithm Experiments

#### 5.2.1. Network Pruning Reconstruction

To test the effect of network pruning reconstruction, experiments are conducted on four real-world network datasets and four artificially generated network datasets. The range of cutoff values used in the experiments is [0, 6], and the pruning reconstruction of the network with different cutoff values, including the number of nodes, the number of connected edges, and the number of connected graphs after reconstruction, are shown in Table 4 and Table 5, where cutoff = 0 corresponds to the original network and cutoff = k means only the edges between nodes with a cutoff ≥ k are retained.

As can be observed from the experiment results, the number of connected sub-graphs is the actual CN when pruning reconstruction is performed for small-scale networks with obvious community structure. For other small-scale networks with complex community structures, the number of connected sub-graphs obtained by pruning reconstruction is related to the average node degree, node degree distribution, and actual community size distribution of the network itself, which can be more easily observed in the experiments of LFR synthetic networks. The node degree distribution and community size distribution of the LFR synthetic network both obey a power-law distribution, such as dataset L1, which has a mixing parameter of 0.3, and its community structure is clearer compared with the network with a mixing parameter of 0.5; its average node degree is 15, and the number of connected sub-graphs obtained by pruning reconstruction at a cutoff value of 5 is 48, which is close to the actual CN 49. For a dataset with a large average degree, such as the L4 dataset, the network still cannot be split at a cutoff value lower than 4, and the number of connected graphs is 1. But accordingly, fewer nodes are removed when pruning such a network, and the obtained connected sub-graphs can preserve more information about the original community structure. In a word, the pruned network is more similar to the real community partition, and as the initial state of the system for Monte Carlo sampling, it will help to reduce the number of iterations required to reach the equilibrium state.

#### 5.2.2. Comparison Results

The following algorithms are selected to compare with our CN estimation algorithm:

- Algorithm A1: the topological potential-based CN estimation algorithm [12].
- Algorithm A2: the statistical inference-based CN estimation algorithm [8].
- Algorithm A3: the statistical inference-based fast CN estimation algorithm [16]

**Figure 8:**
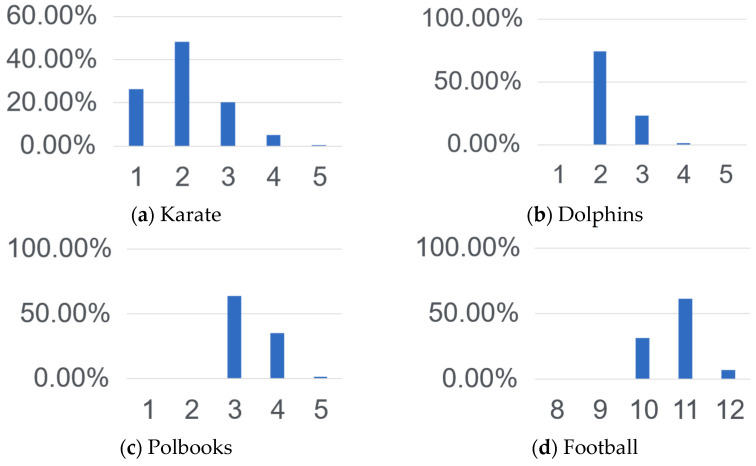
Frequency statistics of ***k*** in BI-CNE.

Ten 10,000-round Monte Carlo sampling experiments are conducted on each of the four real-world network datasets, and the one with the highest average likelihood P(A|g,k) is selected, and the frequency of the number of communities k during their sampling is counted, as shown in Figure 8. The value with the highest frequency, i.e., the k value corresponding to the highest posterior probability P(A|g,k), is taken as the estimated number of communities. The results of the estimation on four real-world networks are shown in Table 6, compared with Algorithms A1, A2, and A3. The estimated number is considered better if it is closer to the ground-truth number of communities K.

It shows that the BI-CNE algorithm is able to exactly estimate the number of real communities on the first three datasets, including Karate, Dolphins, and Polbooks. For the Football dataset, the number of real communities is 12, which contains 11 communities corresponding to 11 soccer federations, and the 12th community is actually the soccer teams that do not belong to any soccer federations. The number of communities estimated by the BI-CNE algorithm is 11, which is the closest to the ground-truth number of communities among the existing community estimation algorithms. The experiments show that the BI-CNE algorithm generally outperforms the other comparative algorithms on real-world networks.

To verify the generalization capability of the BI-CNE algorithm, 10 times 100,000 rounds of Monte Carlo sampling experiments were conducted on each of the LFR synthetic network datasets. In the same way, the k value with the highest frequency was taken as the final estimated number of communities. The results of the comparison on LFR synthetic networks are shown in Table 7.

The experimental results show that Algorithm A1, based on topological potential, can only estimate a small number of communities on a synthetic network of 1000 nodes, which is caused by the lack of a rigorous theoretical basis for the heuristic operation of the algorithm itself and the simple secondary processing of the results. Algorithm A2 cannot converge on the synthetic networks and fails to estimate the number of communities due to its low sampling acceptance rate and slow operation speed. Algorithm A3 estimates a close number on L1, but if the average degree is lower or the mixing parameter is higher, it tends to overestimate the number of communities. That is because the community structure will be more obscure with a lower average degree and a higher mixing parameter.

The BI-CNE algorithm accurately estimates the number of communities on L1 and L2 with the same mixing parameter of 0.3 and obtains the closest results compared to the other algorithms on L3 and L4.

#### 5.2.3. Sampling Acceleration

**Figure 9:**
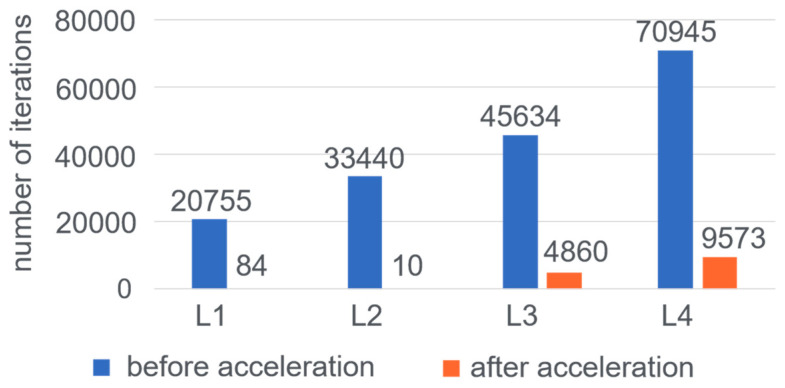
The number of iterations of the convergence process of LFR networks.

To verify the effectiveness of Monte Carlo sampling acceleration, the number of iterations required to converge to a smooth distribution and the sampling acceptance rate during the convergence process are counted separately on the BI-CNE algorithm before and after the acceleration is applied. For statistical convenience, the state of convergence is defined as the final estimated CN being sampled 10 times consecutively. For the LFR synthetic network datasets, a comparison of the number of iterations needed to converge before and after applying acceleration is shown in Figure 9.

Figure 9 shows that the number of iterations needed for convergence increases if the network has a lower average node degree and a higher mixing parameter, which usually means the structure of the network is more obscure. It also shows that after applying the acceleration to sampling, the number of iterations needed is far less, and the algorithm can quickly enter a smooth state for parameter probability sampling. There are two main reasons for the significant acceleration effect. First, the Monte Carlo sampling without acceleration starts with a random initial probability distribution to iterate, while the one with acceleration uses the connected sub-graphs after network pruning and reconstruction as the initial state. The initial probability distribution of the pruned network is closer to the final converged target probability distribution, thereby reducing the number of redundant iterations. Secondly, more effective operations are achieved in each round of sampling after applying the acceleration, which raises the sampling acceptance rate of the operations and makes Monte Carlo sampling reach the smooth state faster.

**Figure 10:**
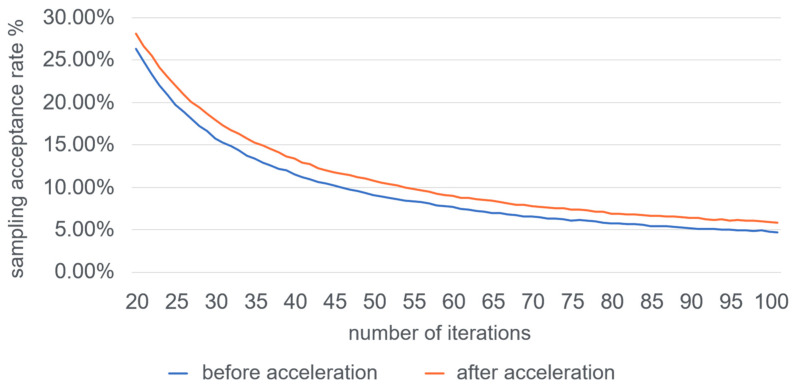
Comparison of sampling acceptance rate before and after acceleration.

In order to observe the improvement of the sampling acceptance rate more intuitively, a sampling acceleration comparison experiment is conducted on a large-scale network dataset, Amazon, which has 334,863 nodes and 925,872 edges. To facilitate the observation, the statistics are counted from the twentieth iteration until the sampling acceptance rate is as low as five percent, and the results are shown in Figure 10.

#### 5.2.4. Complexity Analysis

The time complexity of pruning and reconstructing the network is O(m), where m is the total number of edges in the network. In each iteration of Monte Carlo sampling, the ratio of P(A|g,k) before and after the operation needs to be calculated. To reduce the computational cost, it can be obtained by calculating the change of log P(A|g,k), so as to achieve a time complexity of O(k), where k is the number of communities. If sampling acceleration is performed on Operation 1, the weights between the current community r and all other communities need to be calculated in each iteration of sampling to obtain the probability of selecting a specific community as community s. The time complexity required for this process is also O(k). Therefore, the time complexity of the BI-CNE algorithm is O(Rk) in summary, where R is the number of iterations. In practice, the algorithm runs much faster than other comparative algorithms.

### 5.3. PMIK-SC Algorithm Experiments

#### 5.3.1. *l*-Order Transfer Probability Matrix for Approximating the Infinite-Order One

As mentioned in Section 4.2.2, when the target network is large, the time of calculating the PMI matrix can be reduced by using an l-order transfer probability matrix to approximate the infinite-order one. According to Equation (18), the infinite-order transfer probability matrix is essentially the weighted sum of transition probability matrices from 0-th order to h-th order, where h approaches infinity, and the weight for the h-th order is $e^{-h}$. When h goes large, the weight becomes very small, which means that by setting a threshold l, one can ignore the transfer probability matrices beyond the l-th order to obtain an approximate matrix $P_{a}$ for the infinite-order one.

(33) $$P_{a}=\sum_{h=0}^{l+1}e^{-h}{P_{1}}^{h}\approx \sum_{h=0}^{\infty }e^{-h}{P_{1}}^{h}=P$$

In order to avoid the occurrence of zero values in the cumulative transfer probability matrix, which would result in the inability to compute PMI, we assume that for the (l+1)-th and larger orders, the probability for any node to reach other nodes in the network is equal. Therefore, when computing the (l+1)-th order transition probability matrix, we replace ${P_{1}}^{l+1}$ with a matrix where each element has a value of 1/n, where n is the size of the matrix.

Calculating the matrix $P_{a}$ just requires performing l−1 matrix multiplications. If the Coppersmith–Winograd algorithm [29] is used for matrix multiplication, the time complexity can be reduced to $O(l{\cdot n}^{2.3729})$. In the case of large network scales, replacing the infinite-order transfer probability matrix P with the *l*-th order cumulative transfer probability matrix $P_{a}$ can accelerate the computation of generating the PMI matrix.

In order to analyze the impact of using an l-order transition probability matrix for approximating the infinite-order one, we measure the difference between the PMI matrices produced by the two methods and the corresponding computation time on synthetic networks of different scales.

The difference between the two PMI matrices is quantified using average square error (ASE), defined as:

(34) $$ASE\left(PMI_{1},PMI_{2}\right)=\sum_{i}\sum_{j}{\left[PMI_{1}\left(i,j\right)-PMI_{2}\left(i,j\right)\right]}^{2}/n^{2}$$

where n is the size of the two matrices $PMI_{1}$ and $PMI_{2}$. Table 8 shows the ASE values of the PMI matrix generated by l-order transition probability matrices with different l values and that generated by the infinite-order ones.

The results show that it is feasible to use the l-order transition probability matrix to approximate the infinite-order one. This will introduce a certain error related to l and the network itself. In general, the larger l is, the smaller the error is. When l is large enough, the error can be negligible. For most datasets, choosing l=6 is appropriate.

The impact on the calculation time of using l-order instead of infinite-order is also analyzed. Table 9 shows the time taken to generate a PMI matrix using infinite-order and l-order transition probability matrices.

The results show that, in general, when using an l-order transition probability matrix, more time is needed for more orders. It can also be inferred from the results that using l-order instead of infinite-order has a time advantage only when the scale of the network is large (for example, more than 1000 nodes). The larger the network is, the more obvious the time advantage will be.

#### 5.3.2. Complexity Analysis

First, the algorithm needs to compute the PMI matrix for the given network. An approximate calculation is performed using the l-th order cumulative transfer probability matrix, with a time complexity of $O(l{\cdot n}^{2.3729})$. Then, the PMI is computed using the cumulative transfer probability matrix with a time complexity of $O(n^{2})$. Next is the use of KNN for network reconstruction and Laplacian matrix generation, with complexities of $O(nK_{N})$ and $O(n^{2})$, respectively. In the process of spectral clustering, the complexity of finding the top k eigenvectors of the matrix is $O({kn}^{2})$. The value of k can be set to the estimated maximum possible number of communities. Therefore, the time complexity of clustering using k-means is O(knT). Since l, T, and k are all constants much smaller than n, the overall time complexity of the algorithm is $O(kn^{2})$.

#### 5.3.3. Community Detection Tasks

To evaluate the effectiveness of the PMIK-SC algorithm, we applied it to the community detection task and compared it with multiple benchmark algorithms. Since PMIK-SC is essentially a spectral clustering algorithm, we first incorporate some typical spectral clustering algorithms into the benchmark algorithms, including Comm [30], Heat [20], Katz [31], SCCT [32], and PPR [33]. Secondly, the kernel of PMIK-SC, which measures the relationships between nodes, is generated within the information-theoretic framework. We have also introduced some mutual information-based algorithms, including MINC-NRL [6] and AMI-MLPA [7]. Finally, we have also considered some state-of-the-art community detection algorithms, including the node-embedding-based algorithm GEMSEC [34], Ego-net-based algorithms DEMON [35], and Ego-splitting [36], as well as the motif-enhanced algorithm EdMot [37]. The theoretical time and space complexity of these algorithms are shown in Table 10.

To evaluate the accuracy and efficiency of the PMIK-SC algorithm on community detection, we run the algorithm on real-world networks Karate, Dolphins, Football, Polbooks, and synthetic networks L1, L2, L3, and L4, comparing them with the benchmark algorithms. Table 11, Table 12, Table 13 and Table 14, respectively, show the NMI, modularity, average conductance, and average intra-cluster density of the results, and Table 15 shows the time required for the execution of each algorithm. The bold numbers emphasize the best experimental results within each dataset.

As shown in the above tables, PMIK-SC achieved accurate results in community detection. On average, the partitions obtained by PMIK-SC reach higher NMI and AC than those obtained by the baseline algorithm. Although the performance of PMIK-SC is not as good as that of algorithms such as Ego-Splitting and Edmot in terms of modularity and AICD, it still maintains a high level on both real-world and synthetic networks. It can be inferred from the results that on real-world networks and synthetic networks with small mixing parameters, the results of spectral clustering methods, including Comm, Heat, and Katz, are accurate. However, when the mixing parameter increases, the accuracy of these algorithms drops significantly, except for PMIK-SC and SCCT. When μ=0.4 (which means that the boundaries between a community and the other part of the network are blurred), PMIK-SC can still reach an NMI of 0.990. The accuracy of the algorithms MINC-NRL and AMI-MLPA based on mutual information has also reached a high level, but the accuracy of MINC-NRL on synthetic networks is not perfect. It can be observed from Table 11 that even when μ=0.1 on dataset L1 (which means the community boundaries are very obvious), MINC-NRL can only achieve an NMI of 0.926. This may be caused by MINC-NRL learning some information that is irrelevant to community structure in some dimensions of the embeddings during the process of network representation learning. AMI-MLPA can achieve an NMI of 1.0 on L1, but when μ increases, its accuracy also drops significantly.

As can be inferred from Table 12, when using modularity as the evaluation index, the motif-enhancement-based algorithm EdMot performs best, and the Ego-net-based algorithm Ego-splitting also achieves good results. The average modularity of the results obtained by these two algorithms in the experimental datasets exceeds PMIK-SC, but their NMI is not high. This also prompts us to think about whether modularity is more inclined to consider the local density of the network than NMI, which represents the ground-truth partition. We will conduct further research on this point in the future, trying to introduce some local enhancement mechanisms to improve the performance of modularity for the PMIK-SC algorithm.

In terms of running time, PMIK-SC and other spectral-clustering-based algorithms are also less efficient than local-enhancement-based algorithms like EdMot, DEMON, and Ego-splitting. However, compared with the other spectral clustering algorithms, PMIK-SC is more efficient on larger networks with larger mixing parameters due to the approximate acceleration used in generating PMI kernels and matrix decomposition.

## 6. Conclusions

In this article, a fast Bayesian inference-based number of communities estimation algorithm (BI-CNE) based on a degree-corrected stochastic block model is proposed. The algorithm first prunes and reconstructs the network, then performs Bayesian inference and designs the corresponding Monte Carlo sampling process for obtaining the number of community estimates. At the same time, the connectivity graph results of network pruning and reconstruction are applied to the initial state of sampling, and the overall sampling acceptance rate is improved by controlling the node transfer direction in sampling. Experimental comparisons with other CN estimation algorithms in real-world networks and LFR synthetic networks show that the estimation speed and accuracy of this algorithm outperform other existing CN estimation algorithms. The time complexity of O(Rk) also allows the algorithm to be efficiently extended to large-scale complex networks for community size estimation tasks.

With the premise of obtaining the CN estimation, a new graph kernel based on point-wise mutual information is proposed and applied to the spectral clustering algorithm. Experiments on real-world networks and synthetic networks show that the algorithm has higher accuracy and stability compared with existing community detection algorithms. The comparison results with the current optimal graph-kernel-based spectral clustering algorithm also indicate that the point-wise information kernel can extract network topological information more effectively.

The two algorithms are complementary to community detection. From one point of view, PMIK-SC can reach high accuracy by providing an exact number of communities; from another perspective, taking the number of communities as a priori significantly improves spectral-clustering-based community detection algorithms compared with other approaches.

Due to the symmetry of the PMI matrix, one limitation of the current PMIK-SC algorithm is that it can only be applied to undirected networks. The other limitation is the relatively high time complexity of eigendecomposition, which affects the performance of the PMIK-SC algorithm on large-scale networks.

In future research, we will try to construct an asymmetric proximity matrix based on the PMI matrix to solve the problem of community detection on directed graphs. We will also consider improving the pruning judgment, the re-merging of communities after pruning, and trying to introduce some local enhancement mechanisms to further reduce the problem scale for higher efficiency.

## Figures and Tables

**Table 1:** Main properties of real-world networks.

Dataset	*n*	*m*	*K*
Karate	34	78	2
Dolphins	62	162	2
Polbooks	105	441	3
Football	115	613	12

**Table 2:** Main properties of LFR synthetic networks.

Dataset	*n*	*m*	*K*	*d*	*µ*
L1	1000	7395	51	15	0.1
L2	1000	7646	47	15	0.2
L3	1000	7692	54	15	0.3
L4	1000	7549	44	15	0.4
L5	500	14,086	3	30	0.3
L6	1000	30,288	7	30	0.3
L7	2000	60,306	4	30	0.3
L8	5000	143,972	17	30	0.3
L9	10,000	302,282	87	30	0.3

**Table 3:** Hardware Information.

CPU	Intel(R) Core(TM) i7-9700
Cores	8
Frequency	3.0 GHz
Memory	8 GB

**Table 4:** Reconstruction results of real-world networks.

	Karate			Dolphins			Polbooks			Football		
Cutoff	Node	Edge	Part	Node	Edge	Part	Node	Edge	Part	Node	Edge	Part
0	34	78	1	62	159	1	105	441	1	115	613	1
1	32	67	1	46	121	1	104	423	1	115	517	1
2	17	32	1	40	84	2	98	364	1	115	449	2
3	11	18	2	25	45	4	84	289	4	113	411	8
4	6	7	2	16	21	3	65	221	4	108	393	10
5	6	4	2	9	8	3	48	128	4	105	327	13
6	4	2	2	8	5	3	33	81	2	95	219	18

**Table 5:** Reconstruction results of LFR synthetic networks.

	L1			L2			L3			L4		
Cutoff	Node	Edge	Part	Node	Edge	Part	Node	Edge	Part	Node	Edge	Part
0	1000	7652	1	1000	15,354	1	1000	7603	1	1000	15,078	1
1	1000	5899	9	1000	12,688	1	994	4835	1	1000	11,559	1
2	999	5410	28	1000	11,281	1	951	3576	10	1000	8918	1
3	995	5131	36	1000	10,840	5	814	2667	25	1000	7650	1
4	964	4656	46	1000	10,759	22	606	1845	38	998	6919	2
5	791	3749	48	1000	10,720	27	472	1309	41	996	6290	15
6	658	3057	42	1000	10,654	29	367	962	37	977	5564	29

**Table 6:** Number of communities estimated on real-world networks.

Dataset	K	A1	A2	A3	BI-CNE
Karate	2	2	2	2	2
Dolphins	2	2	2	3	2
Polbooks	3	4	5	5	3
Football	12	10	11	11	11

**Table 7:** Number of communities estimated on LFR networks.

Dataset	K	A1	A2	A3	BI-CNE
L1	49	11	-	47	49
L2	29	6	-	54	29
L3	49	11	-	72	63
L4	31	8	-	80	51

**Table 8:** ASE of the PMI matrices generated by the two methods.

Orders (*l*)	L5	L6	L7	L8	L9
1	0.038	0.035	0.035	0.010	0.031
2	0.012	0.024	0.011	0.004	0.022
3	0.004	0.014	0.001	0.008	0.007
4	0.001	0.008	0.000	0.009	0.002
5	0.000	0.002	0.000	0.003	0.000
6	0.000	0.000	0.000	0.001	0.000
7	0.000	0.000	0.000	0.000	0.000
8	0.000	0.000	0.000	0.000	0.000
9	0.000	0.000	0.000	0.000	0.000

**Table 9:** Time spent (seconds) to generate a PMI matrix.

Orders (*l*)	L5	L6	L7	L8	L9
infinite	0.606	2.389	9.843	61.675	272.757
1	2.750	2.722	3.109	8.035	36.333
2	2.758	2.765	3.201	9.465	49.704
3	2.773	2.758	3.332	9.992	51.951
4	2.779	2.789	3.371	10.816	56.247
5	2.777	2.844	3.424	12.063	63.485
6	2.792	2.883	3.538	13.263	75.159
7	2.729	2.765	3.662	14.160	82.486
8	2.815	2.851	3.724	15.348	86.140
9	2.830	2.875	3.725	16.142	92.631

**Table 10:** Time and space complexity of the algorithms.

Algorithm	Time Complexity	Space Complexity	
PMIK-SC	$O(kn^{2})$	$O(n^{2})$	n is the number of nodes of the network.
Comm	$O(n^{3})$	$O(n^{2})$	
Heat	$O(n^{3})$	$O(n^{2})$	
Katz	$O(n^{3})$	$O(n^{2})$	
SCCT	$O(n^{3})$	$O(n^{2})$	
PPR	$O(n^{3})$	$O(n^{2})$	
MINC-NRL	$O({kn}^{2})$	O(nL)	k is the max number of communities. L is the max number of levels in overlapping hierarchical clustering.
AMI-MLPA	$O({nk}^{2})$	O(np)	p is the max number of the labels for each node.
GEMSEC	O(n)	O(nq)	q is the dimension of the embeddings for each node.
EdMot	$O(m^{1.5}+n\log\;n)$	$O(n^{2})$	m is the number of edges of the network.
DEMON	O(n)	O(nd)	d is the average degree of the nodes
Ego-splitting	$O(m^{\frac{3}{2}})$	O(nd)	

**Table 11:** NMI of PMIK-SC results compared with the other algorithms.

	Karate	Dolphins	Football	Polbooks	L1	L2	L3	L4	Avg.
PMIK-SC	**1.000**	**0.889**	0.924	0.589	0.999	**0.997**	**0.994**	**0.990**	**0.923**
Comm	0.836	0.655	0.728	0.360	0.873	0.816	0.755	0.662	0.711
Heat	0.836	**0.889**	0.924	**0.601**	**1.000**	0.913	0.869	0.630	0.833
Katz	**1.000**	0.704	0.718	0.319	0.835	0.786	0.716	0.616	0.712
SCCT	**1.000**	**0.889**	**0.927**	0.563	**1.000**	0.982	0.963	0.961	0.911
PPR	0.580	0.407	0.731	0.424	0.808	0.737	0.700	0.575	0.620
MINC-NRL	**1.000**	**0.889**	**0.927**	0.523	0.926	0.886	0.804	0.122	0.760
AMI-MLPA	**1.000**	**0.889**	0.924	0.545	**1.000**	0.999	0.984	0.815	0.895
GEMSEC	0.442	0.337	0.822	0.408	0.473	0.35	0.315	0.305	0.432
Edmot	0.579	0.493	0.904	0.472	0.995	0.981	0.965	0.974	0.795
DEMON	0.244	0.362	0.632	0.466	0.989	0.905	0.860	0.792	0.656
Ego-splitting	0.545	0.496	0.911	0.500	0.995	0.981	0.963	0.974	0.796

**Table 12:** Modularity of PMIK-SC results compared with the other algorithms.

	Karate	Dolphins	Football	Polbooks	L1	L2	L3	L4	Avg.
PMIK-SC	0.371	0.379	0.601	0.496	0.874	0.759	0.655	0.563	0.587
Comm	0.345	0.377	0.456	0.432	0.7	0.554	0.431	0.321	0.452
Heat	0.360	0.379	0.601	0.485	0.875	0.67	0.519	0.279	0.521
Katz	0.371	0.364	0.438	0.382	0.652	0.529	0.373	0.278	0.423
SCCT	0.371	0.379	0.601	0.502	0.875	0.751	0.619	0.541	0.580
PPR	0.334	0.289	0.426	0.402	0.609	0.405	0.339	0.234	0.380
MINC-NRL	0.371	0.379	0.601	0.46	0.872	0.759	0.654	0.069	0.521
AMI-MLPA	0.371	0.379	0.601	0.493	0.875	0.762	0.633	0.436	0.569
GEMSEC	0.436	0.495	0.583	0.543	0.458	0.349	0.305	0.211	0.423
Edmot	**0.514**	**0.602**	0.650	**0.579**	**0.887**	**0.787**	**0.702**	**0.615**	**0.667**
DEMON	0.130	0.311	0.450	0.390	0.852	0.578	0.44	0.301	0.432
Ego-splitting	0.502	0.598	**0.651**	0.565	**0.887**	**0.787**	**0.702**	**0.615**	0.663

**Table 13:** AC of PMIK-SC results compared with the other algorithms (smaller values are better).

	Karate	Dolphins	Football	Polbooks	L1	L2	L3	L4	Avg.
PMIK-SC	**0.132**	**0.064**	0.337	**0.122**	0.104	0.213	0.312	0.404	**0.211**
Comm	0.162	0.103	0.518	0.240	0.396	0.493	0.589	0.667	0.396
Heat	0.152	**0.064**	0.337	0.151	0.102	0.319	0.426	0.643	0.274
Katz	**0.132**	0.102	0.519	0.280	0.428	0.534	0.631	0.702	0.416
SCCT	**0.132**	0.064	0.340	0.143	0.102	0.280	0.383	0.447	0.236
PPR	0.182	0.175	0.543	0.285	0.438	0.578	0.642	0.754	0.450
MINC-NRL	**0.132**	**0.064**	0.340	0.246	0.088	0.176	**0.254**	0.445	0.218
AMI-MLPA	**0.132**	**0.064**	0.337	0.163	0.102	0.203	0.433	0.643	0.260
GEMSEC	0.428	0.354	0.283	0.332	0.090	0.299	0.386	0.463	0.329
Edmot	0.270	0.230	**0.263**	0.254	**0.088**	**0.172**	0.257	**0.344**	0.235
DEMON	0.802	0.840	0.316	0.584	0.112	0.287	0.415	0.566	0.490
Ego-splitting	0.296	0.233	**0.263**	0.255	**0.088**	**0.172**	0.257	**0.344**	0.239

**Table 14:** AICD of PMIK-SC results compared with the other algorithms.

	Karate	Dolphins	Football	Polbooks	L1	L2	L3	L4	Avg.
PMIK-SC	0.252	0.171	0.848	0.270	0.707	0.568	0.584	0.451	0.481
Comm	0.250	0.155	0.624	0.209	0.628	0.490	0.443	0.336	0.392
Heat	0.250	0.171	0.848	0.259	0.710	0.581	0.567	0.479	0.483
Katz	0.252	0.169	0.621	0.239	0.542	0.416	0.382	0.267	0.361
SCCT	0.252	0.171	**0.860**	0.240	**0.710**	0.486	0.496	0.398	0.452
PPR	0.251	0.192	0.594	0.211	0.585	0.428	0.396	0.238	0.362
MINC-NRL	0.252	0.171	**0.860**	0.176	0.558	0.463	0.412	0.290	0.398
AMI-MLPA	0.252	0.171	0.848	0.226	**0.710**	0.582	0.491	0.175	0.432
GEMSEC	**0.626**	**0.609**	0.841	**0.616**	0.459	**0.626**	**0.620**	**0.556**	**0.619**
Edmot	0.526	0.393	0.828	0.450	0.665	0.467	0.438	0.347	0.514
DEMON	0.144	0.052	0.316	0.083	0.699	0.516	0.465	0.267	0.318
Ego-splitting	0.464	0.387	0.817	0.546	0.667	0.467	0.426	0.347	0.515

**Table 15:** Time usage of PMIK-SC compared with the other algorithms.

	Karate	Dolphins	Football	Polbooks	L1	L2	L3	L4
PMIK-SC	9.337	5.641	6.077	6.219	12.660	13.203	12.649	13.417
Comm	0.439	0.058	1.737	0.699	19.188	20.465	21.590	18.870
Heat	0.487	0.075	0.904	0.637	17.420	18.511	22.907	22.231
Katz	0.554	0.069	0.405	0.090	15.766	14.057	17.949	13.426
SCCT	0.647	0.083	0.298	0.203	16.246	32.359	31.506	37.175
PPR	0.461	0.647	2.268	2.033	19.787	19.341	21.555	19.339
MINC-NRL	0.138	0.245	0.687	0.482	11.137	11.474	10.910	12.077
AMI-MLPA	0.172	0.618	14.705	0.069	4.506	8.490	52.197	148.010
GEMSEC	13.564	26.322	48.974	42.578	349.881	298.914	297.126	297.421
Edmot	0.022	0.005	0.010	0.010	0.126	0.150	0.180	0.149
DEMON	0.018	0.019	0.076	0.049	1.649	1.166	1.000	0.871
Ego-splitting	0.030	0.006	0.018	0.014	0.153	0.193	0.187	0.161

## Data Availability

The datasets generated and analyzed during the current study are available from the corresponding author upon reasonable request.
